# Optimization of the Structural Performance of Buried Reinforced Concrete Pipelines in Cohesionless Soils

**DOI:** 10.3390/ma15124051

**Published:** 2022-06-07

**Authors:** Odey Alshboul, Ghassan Almasabha, Ali Shehadeh, Omar Al Hattamleh, Ali Saeed Almuflih

**Affiliations:** 1 Department of Civil Engineering, Faculty of Engineering, The Hashemite University, P.O. Box 330127, Zarqa 13133, Jordan; ghassans@hu.edu.jo (G.A.); hattam@hu.edu.jo (O.A.H.); 2 Department of Civil Engineering, Hijjawi Faculty for Engineering Technology, Yarmouk University, P.O. Box 566, Irbid 21163, Jordan; ali.shehadeh@yu.edu.jo; 3 Department of Industrial Engineering, King Khalid University, Fahad St, Guraiger, Abha 62529, Saudi Arabia; asalmuflih@kku.edu.sa

**Keywords:** reinforced concrete pipelines, optimum diameter-to-thickness ratio, buried pipelines, deep embankment soil

## Abstract

Pipelines are widely used to transport water, wastewater, and energy products. However, the recently published American Society of Civil Engineers report revealed that the USA drinking water infrastructure is deficient, where 12,000 miles of pipelines have deteriorated. This would require substantial financial investment to rebuild. Furthermore, the current pipeline design practice lacks the guideline to obtain the optimum steel reinforcement and pipeline geometry. Therefore, the current study aimed to fill this gap and help the pipeline designers and practitioners select the most economical reinforced concrete pipelines with optimum steel reinforcement while satisfying the shear stresses demand and serviceability limitations. Experimental testing is considered uneconomical and impractical for measuring the performance of pipelines under a high soil fill depth. Therefore, a parametric study was carried out for reinforced concrete pipes with various diameters buried under soil fill depths using a reliable finite element analysis to execute this investigation. The deflection range of the investigated reinforced concrete pipelines was between 0.5 to 13 mm. This indicates that the finite element analysis carefully selected the pipeline thickness, required flexural steel reinforcement, and concrete crack width while the pipeline does not undergo excessive deformation. This study revealed that the recommended optimum reinforced concrete pipeline diameter-to-thickness ratio, which is highly sensitive to the soil fill depth, is 6.0, 4.6, 4.2, and 3.8 for soil fill depths of 9.1, 12.2, 15.2, and 18.3 m, respectively. Moreover, the parametric study results offered an equation to estimate the optimum pipeline diameter-to-thickness ratio via a design example. The current research outcomes are imperative for decision-makers to accurately evaluate the structural performance of buried reinforced concrete pipelines.

## 1. Introduction

Pipelines are widely used in infrastructures to transport water, wastewater, and energy products. The USA uses 2.2 million miles of underground pipes to deliver drinking water. However, the recently released report by ASCE [1] classifies the USA drinking water infrastructure as a poor category “-C”, as the pipelines are aged. Financing shortages result in an estimated water leakage of 6 billion gallons per day of drinking water. The estimated deteriorated pipelines length was 12,000 miles in 2019 [1]. Pipelines and culverts are considered the most economical options compared with highway bridges, where the utilities of water, wastewater, or energy products intersect with roadways and barriers of high soil depths. However, limited studies were conducted to investigate the performance of pipelines and culverts under deep soil burial since it is difficult to measure the response of such structures experimentally, and it costs a significant amount of money to conduct the experimental setup. On the other hand, the successful use of validated finite element analysis in recent years has significantly helped researchers and designers to measure the structural response of such infrastructural utilities.

The interaction between the pipeline structure and the compacted soil around them is critical in their structural performance. The interface layer between the pipeline structure and the surrounding soil requires a unique estimation. Analyzing such a soil−structure interaction is not easy to estimate, as no closed-form solution can predict this interaction accurately; however, this interaction can be effectively analyzed using the finite element analysis (FEA) [2,3,4,5,6]. In addition, the soil−structure interaction usually is not considered in pipeline and box culverts designs, where the applied pressure above the pipeline and the box culvert is assumed to be equivalent to the geostatic load, which is the earth prism weight measured above the pipeline or the box culvert (Pimentel et al., 2009 [7]). As the soil−structure interaction is challenging to experimentally estimate, several studies have efficiently exploited numerical tools such as finite element modeling and artificial intelligence to predict the effect of the soil−structure interaction for various structural types [3,4,5,6,7,8,9,10,11,12,13,14,15,16,17,18,19]. Therefore, the soil−structural interaction should be included in the pipeline and culvert design in order to estimate the structural response of such infrastructures accurately. Excluding this interaction may result in the erroneous structural design of these elements.

Various studies have investigated pipelines’ stress redistribution and performance under a deep soil fill depth. For example, Marston and Anderson (1913) [20] were the pioneer researchers who studied loading distribution on buried pipes, whereas Marston (1930) [21] found that installation conditions and pipe properties influence the loading distribution. Likewise, Spangler (1950 [22], 1968 [23]) revealed that vertical pressure on the steel buried pipes developed by the surrounding compacted soil layers is coupled to the relative settlement between the soil barrier and the original soil. Similarly, Abuhajar et al. (2015) [24] indicated that soil arching is significantly affected by the soil fill depth, culvert thickness, elastic modulus of soil, and poison ratio.

Bashir (2000) [25] investigated several steel pipelines with diameter-to-thickness ratios ranging from 50 to 150, subject to soil fill depths ranging from 0.6 m to 3.7 m. The study detected that regardless of the pipe diameter-to-thickness ratio, the live load of vehicles was insignificant for soil depths more than 2.5 m. Similarly, Orton et al. (2015) [26] investigated 10 reinforced concrete box culverts, and the soil fill depth ranged from 0.76 to 4.1 m. The study found that the live load of vehicles was not significant when the soil fill depth increased. In addition, the experimental results indicate that the deflection of culverts subjected to soil fill of less than 2.4 m is overestimated by the American Association of State Highway and Transportation Officials (AASHTO) load resistance design factors (LRFD) bridge design specifications (AASHTO 2012 [27]).

The experimental investigation of the structural performance of pipelines and culverts under a high soil fill depth is uneconomical and difficult to conduct. Finite element modeling is an effective alternative tool. For instance, Shatnawi et al. (2017) [28] effectively used the finite element analysis (CANDE software) to explore the behavior of various box culvert geometries under a high soil fill depth. This paper used CANDE-2019 [29] as the FEA tool to perform the parametric study.

In this study, FEA was used to investigate the structural performance of reinforced concrete pipelines under various soil fill depths (9.1, 12.2, 15.2, and 18.3 m) with different pipe diameters (610, 915, 1220, and 1524 mm). A soil depth of 18 m may be reached in deep embankment soil; an example of deep-buried culverts is the Peter Smith Brook Culvert with a 23.4 m deep embankment soil, which is a part of a highway upgrade near Longs Creek, New Brunswick, Canada [30]. This study provides a guideline for engineers to estimate the most efficient pipeline geometry for various soil filling depths. In addition, this research also explored the optimum diameter-to-thickness ratio for various pipeline conditions. The shear reinforcement in this study was excluded, as Yee (2003) [31] found that the provided shear reinforcement in reinforced concrete culverts did not improve the load capacity of the culverts, but the culvert section became over-reinforced. Shatnawi et al., 2017 [28], previously published work related to the investigated topic, and thus this study aimed to compare the obtained results with the previously gained ones.

## 2. Methodology

Using finite element analysis (FEA), this study investigated reinforced concrete (RC) buried pipelines under various soil depths using finite element analysis (FEA). Culvert Analysis and Design (CANDE-2019 software [29]) was used to conduct the nonlinear FEA. The description of CANDE-2019 [29] is discussed in Section 3.

**Figure 1:**
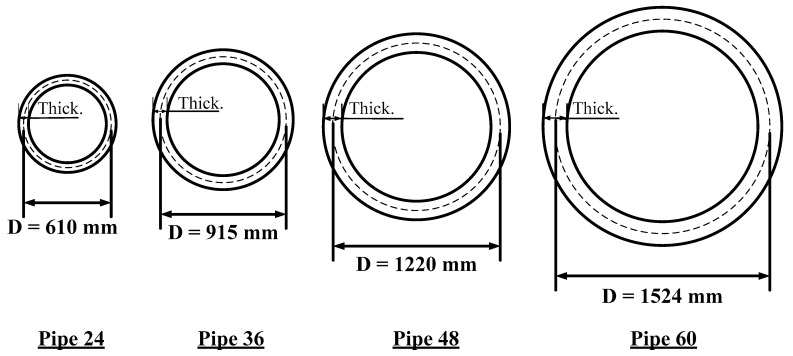
Geometric dimensions of the investigated pipeline culverts in this study.

The following points represent the most critical features of the executed FEA modeling. Various pipe diameters, as shown in Table 1, were investigated, where Pipe 24, Pipe 36, Pipe 48, and Pipe 60 were 610, 915, 1220, and 1524 mm, respectively. The geometric properties of the RC pipelines are illustrated in Figure 1.

- Varying soil fill depths (h) were investigated: 9.1, 12.2, 15.2, and 18.3 m.
- Flexural steel reinforcement (FSR) was provided in the RC pipelines. However, no shear reinforcement was provided.
- Duncan et al. (1980 [32]) and Selig (1988 [33])’s hyperbolic stress−strain parameters were used for the nonlinear FEA soil properties.
- The parametric FEA study investigated various variables (e.g., soil fill depth, pipeline diameter, and pipeline thickness).

Fifty-eight FEA runs were executed to calculate the FSR for each pipeline model, as indicated in Table 2. In each FEA model run, the following process was carried out:

1. The soil fill depth was defined considering the values in Table 2.
2. The diameter of the RC pipeline was selected from the dimensions specified in Table 1.
3. The thickness of the RC pipeline was reduced in each FEA model until the ratio of applied-to-capacity shear stress (τ_a_/τ_c_) was equal to or less than the unity to exclude the shear reinforcement and reduce the cost of constructing the pipelines. In addition, the thickness reduction process was stopped once the maximum crack width (*w_max_*) reached 0.25 mm, which allowed the pipeline to have small crack width at the ultimate loading state.
4. The FEA results were summarized and analyzed to investigate the effect of soil depth on the diameter and thickness of the reinforced concrete pipeline.
5. The total required FSR and the maximum deflection (∆_max_) of each pipe were recorded.

**Figure 2:**
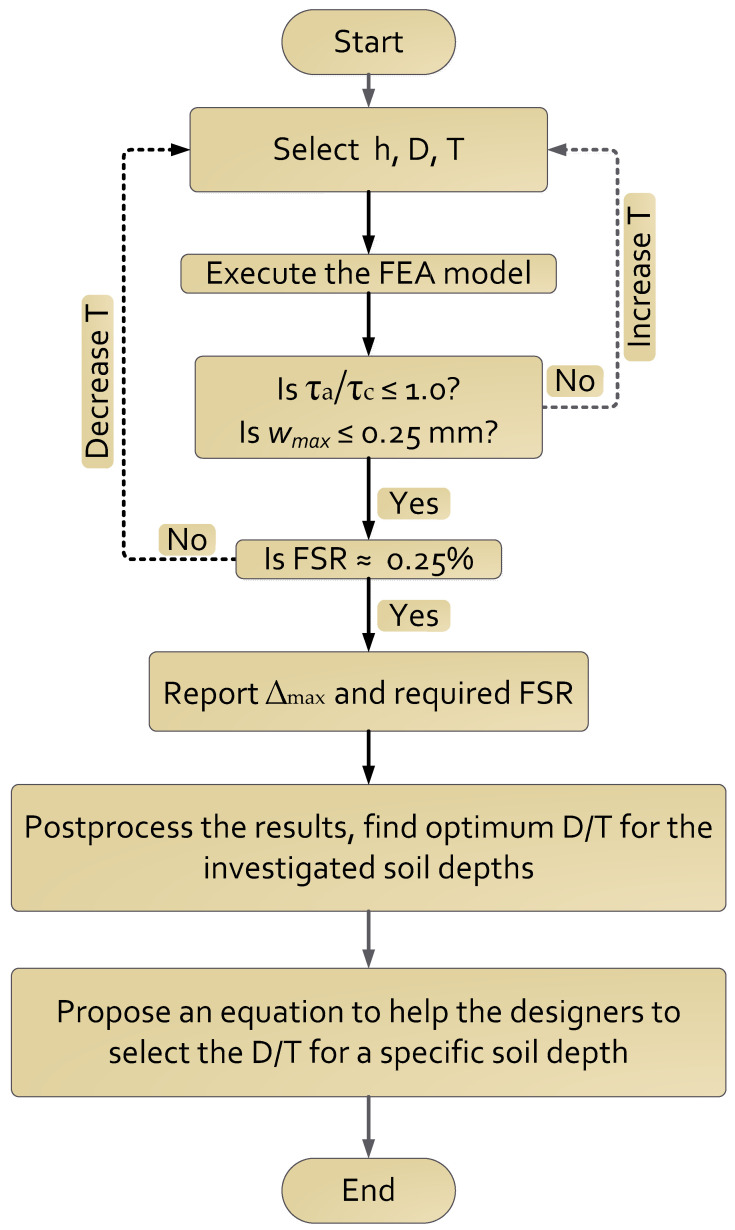
Flow chart to summarize the methodology of this study.

As discussed in Step 3 in the procedure mentioned above, the shear reinforcement was omitted. Furthermore, it was excluded because the ratio of applied-to-capacity shear stress was equal to or less than unity. The flowchart summary of the methodology in this study is shown in Figure 2.

## 3. FEA Model and Construction Stages

**Figure 3:**
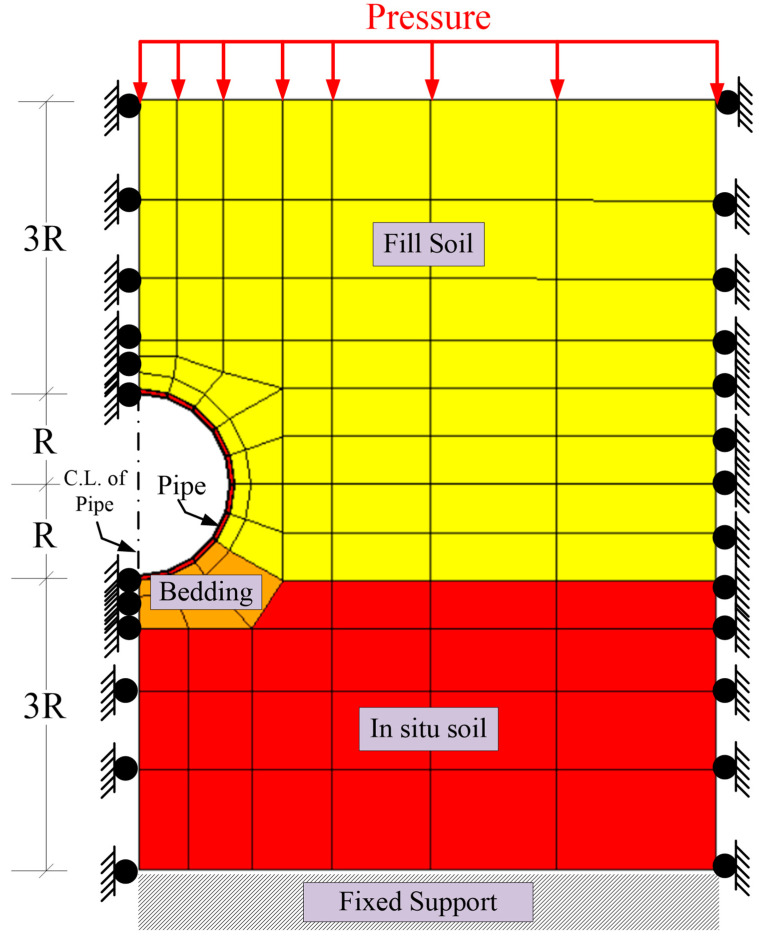
FEA model soil layers, boundary conditions, and loading pressure.

Figure 3 represents the FEA model’s geometry, boundary conditions, and mesh. The soil layers are the in situ soil, bedding, and fill soil. As the pipeline is symmetric about the pipe centerline, one-half of the pipe and soil layers were modeled. The pipeline was simulated using 10 equal-length beam elements connected between 11 nodes. The 2D quadrilateral elements of the modeled soil layers were defined below and above the pipeline up to three times the pipeline radius. The load factor of 1.42 was defined according to AASHTO 2020 [34]. Therefore, the load pressure equals the factor 1.42 multiplied by the soil fill unit weight and soil fill depth. The live load was excluded from the FEA as all of the investigated cases in this study were subjected to a soil depth more than the 2.44 m-threshold proposed by AASHTO 2020 [34], as the effect of live load on culverts diminishes for soil depths of more than 2.44 m. The bottom layer of the in situ soil was restrained in the horizontal and vertical directions.

In contrast, the left and right sides of the model were restrained in the horizontal direction only because the model was symmetric at the pipeline’s centerline. The FEA model considered the soil−structure interaction, as shown in Figure 3. It is worth mentioning that an extensive investigation of the literature was conducted. However, the literature lacked experimental results related to this study. On the other hand, the used model was reliable and was validated using the project developed under the National Cooperative Highway Research Project NCHRP 15–28 [35]. In this study, the Level 2 option (automatic FEA) automatically created the nodes and the optimum mesh size of the FEA model using simple input parameters; this option made the modeling process easy to implement and reduced the required time to generate debug for the mesh of the FEA models. The automatic mesh selected coarse mesh size in the soil layers and fine mesh size in the pipeline geometry, as we were more concerned about the response of the pipeline.

**Figure 4:**
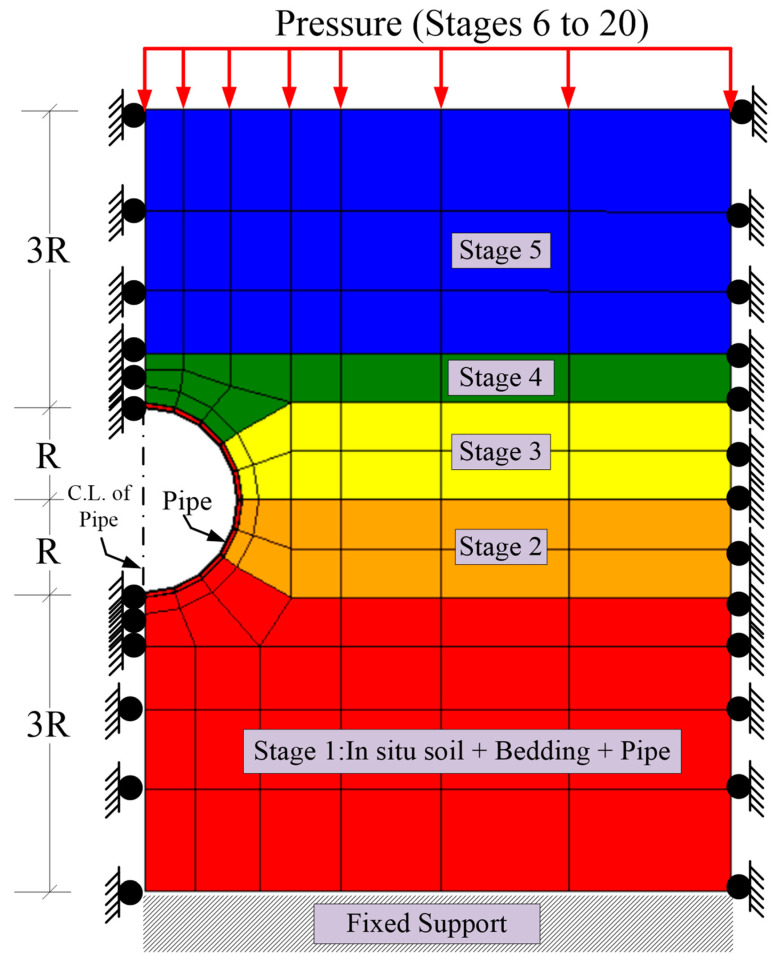
Construction and pressure stages of the FEA model.

It is imperative to simulate the construction stages of the reinforced concrete pipelines in order to model the structural responses of the pipelines accurately. Therefore, the pipeline, bedding, and in situ soil layer were modeled in the first stage, while the soil fill was added gradually in the second, third, fourth, and fourth stages, respectively. On the other hand, to increase the analysis accuracy and avoid solution divergence, the loading pressure was divided into several equal increments applied to the model in stages 6 to 20, as shown in Figure 4.

### 3.1. FEA Soil Properties

We utilized three types of soil layers in the FEA model. The Duncan/Selig parameters were proposed by Duncan et al. (1980) [32] and were modified by Selig (1988) [33], where SW95 and SW90 gravely sand with 95% compaction was used to define the FEA modeling. The SW95 and SW90 soil were used in the fill soil and bedding. The library of CANDE-2019 contains the hyperbolic stress−strain parameters of SW95 and SW90, as summarized in Table 3 [36]. The in situ soil was considered isotropic, where the soil’s young’s modulus was 34.5 MPa, Poisson’s ratio was 0.4, and soil density was 18.9 kg/m^3^. CANDE-2019 offers various culvert installations, including barrier and trench installations. In general, the installation type refers to the location of the culvert relative to the ground level. In the embankment installation, the bottom face of the culvert coincides with the original ground level, where soil excavation is not needed. While trench installation refers to the case when the bottom face of the culvert is below the original soil level, in this case, soil excavation is needed. In this study, embankment installation was considered in the FEA modeling.

### 3.2. FEA Steel and Concrete Properties

Normal strength concrete with a compressive stress of 34.5 MPa and unit weight of 23.6 kg/m^3^ was utilized, the 448 MPa tensile strength steel reinforcement with a concrete cover of 32 mm was used, and more details of the concrete and steel properties are summarized in Table 4.

## 4. FEA Results and Discussions

The parametric study was conducted using CANDE 2019 software. The deformation response and required flexural steel reinforcement (FSR) were recorded and analyzed. The following two sections illustrate the FEA modeling outputs and the detailed analysis.

### 4.1. Deformed FEA Model

**Figure 5:**
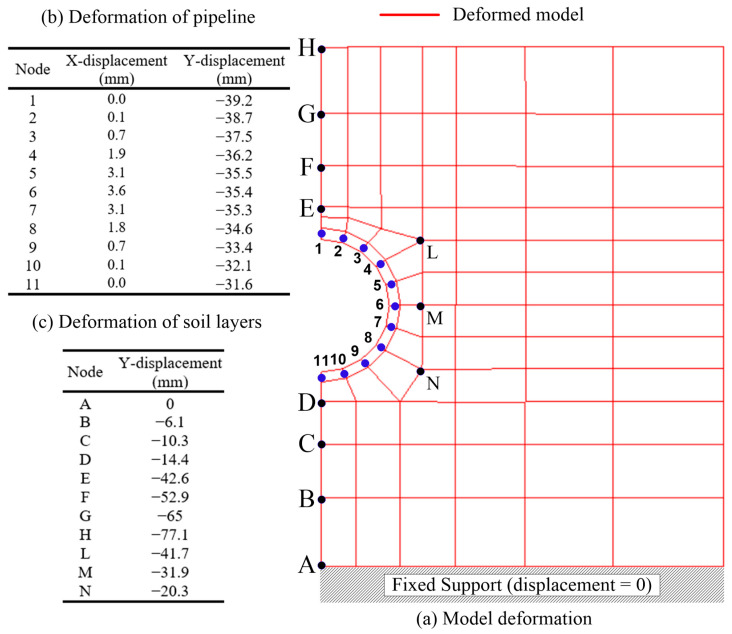
Sample of FEA model results for a soil fill depth of 18.3 m, diameter of 1524 mm, and pipe thickness of 152 mm: (**a**) deformed model, which includes the pipeline elements with nodes 1 to 11 and selected soil nodes A to N, (**b**) nodal displacement of the pipeline structural elements, and (**c**) Y-displacement of selected soil nodes.

Figure 5 shows the FEA model’s deformed shape representing a reinforced concrete pipeline with a diameter of 1524 mm, pipeline thickness of 152 mm, and soil fill depth of 18.3 m. Nodes A, B, C, D, E, F, G, H, L, M, and N represent various in situ soil, bedding, and soil fill, while points 1 to 11 connect the pipeline elements. The vertical displacement (Y-displacement) of points A, B, C, and D was 0, −6.1, −10.3, and −14.4 mm, respectively. It is worth mentioning that the vertical displacement was cumulative, e.g., the vertical displacement at point C was equal to the vertical displacement at point B plus the local deflection at point B, which resulted from the stress demand at point C. Similarly, the net deflection that occurred in the pipe can be calculated using the relative displacement between points 1 and 11, which represent the extreme top and bottom points of the pipeline. In the case of Figure 5, the pipeline experienced a deflection of −39.2 mm − (−31.6 mm) = −7.6 mm. The summary of the vertical displacement for points A to N and 1 to 11 are tabulated in Figure 5.

**Figure 6:**
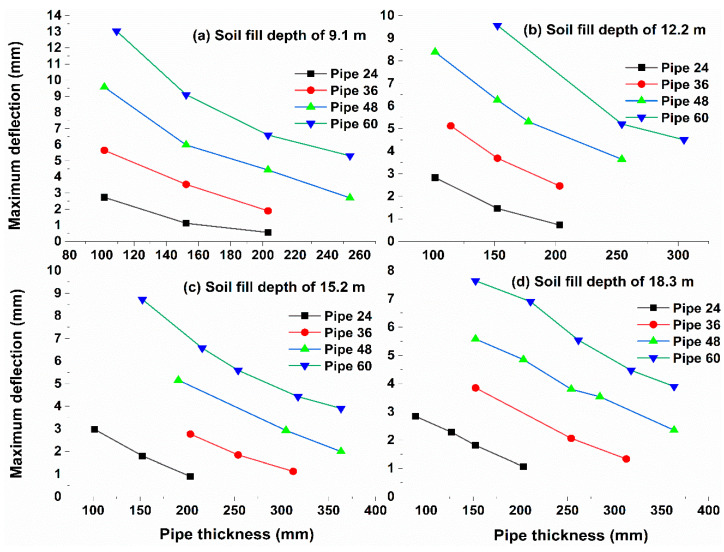
Maximum deflection versus reinforced concrete pipe thickness for various soil fill depths.

The pipeline deflection for the investigated parametric study can be plotted using the procedure mentioned above, as shown in Figure 6, for soil fill depths of 9.1, 12.2, 15.2, and 18.3 m. Figure 6a shows that if Pipe 60 with a thickness of 254 mm was decreased to 203 mm, 152 mm, or 109 mm, the pipeline deflection increased from 5.5 mm to 6.3 mm, 9.2 mm, or 13 mm, respectively. The same pipeline response trend was noticeable for Pipe 24, Pipe 36, and Pipe 60. The FEA model considers three different variables during the analysis process: pipeline deflection, maximum crack width, and FSR. The maximum crack width must not exceed the 0.25 mm limit, and if this limitation cannot be achieved, the model increases the required FSR, which reduces the crack width and pipeline deflection.

### 4.2. Flexural Steel Reinforcement (FSR)

**Figure 7:**
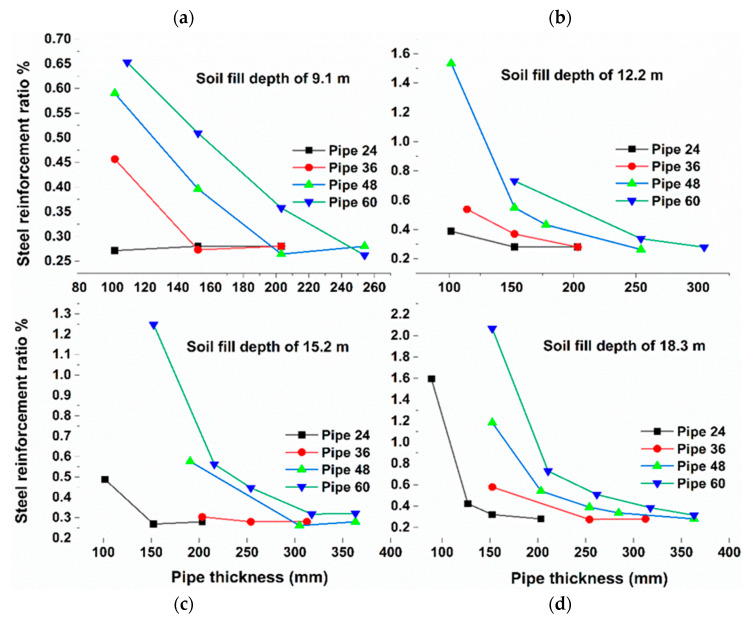
Required steel ratio versus reinforced concrete pipe thickness for various soil fill depths.

The parametric study was performed using CANDE-2019 according to the procedure described in the Methodology. A total of 58 FEA model runs were conducted, as specified in Table 2. In each model run, the required FSR and maximum deflection were reported. Figure 7 illustrates the required FSR corresponding to various reinforced concrete pipeline thicknesses for the soil depths of 9.1, 12.2, 15.2, and 18.3 m. Figure 7a shows the effect of reducing the pipe thickness on the FSR for soil fill depths of 9.1 m. In contrast, the required FSR for Pipe 60 with a cross-section thickness of 254 mm required an FSR of 0.26%; however, if the cross-section of Pipe 60 was reduced to 203, 152, and 109 mm, the required FSR increased to 0.35%, 0.5%, and 0.65%, respectively. Therefore, considering that the most expensive component of the pipe is the steel reinforcement, the optimum thickness of Pipe 60 under soil fill of 9.1 m was 254 mm, as it requires the least amount of steel reinforcement.

Similarly, for Pipe 48, changing the pipe thickness from 254 to 203, 152, or 102 mm, increased the required FSR to 0.26%, 0.39%, and 0.59%, respectively. As a thickness of 203 and 254 mm required the same amount of reinforcement (FSR = 0.26%), the thickness of 203 mm was considered to be optimum, as it reduced the amount of RC. The same criteria can be applied to Pipe 36 and Pipe 24, where the optimum thicknesses that required the least amount of steel reinforcement were 152 and 102 mm, respectively. The same procedure was applied for soil fill depths of 12.2, 15.2, and 18.3 m, as illustrated in Figure 7b–d. The summary of the optimum thickness for all cases is listed in Table 5.

### 4.3. Optimum RC Pipeline Diameter-to-Thickness Ratio

In Section 4.2, identifying the optimum pipeline thickness is illustrated. The summary of the optimum pipeline thickness for various investigated pipeline diameters and soil fills is listed in Table 5. Moreover, the normalized optimum diameter-to-thickness (D/T) ratios from Table 5 were calculated and are listed in Table 6. For example, the optimum thickness of Pipe 24 under a soil fill depth of 9.1 m was 102 mm. Thus, the optimum D/T is 6.0. This procedure was performed for the remaining pipeline geometries and soil fill depths, as shown in Table 6. The last column in Table 6 lists the average optimum D/T ratios for various pipeline diameters for soil fill depths of 9.1, 12.2, 15.2, and 18.3 m. For instance, the average optimum D/T at a soil depth of 15.2 m is the average of 4, 4.5, 4, and 4.2, which equals 4.2.

**Figure 8:**
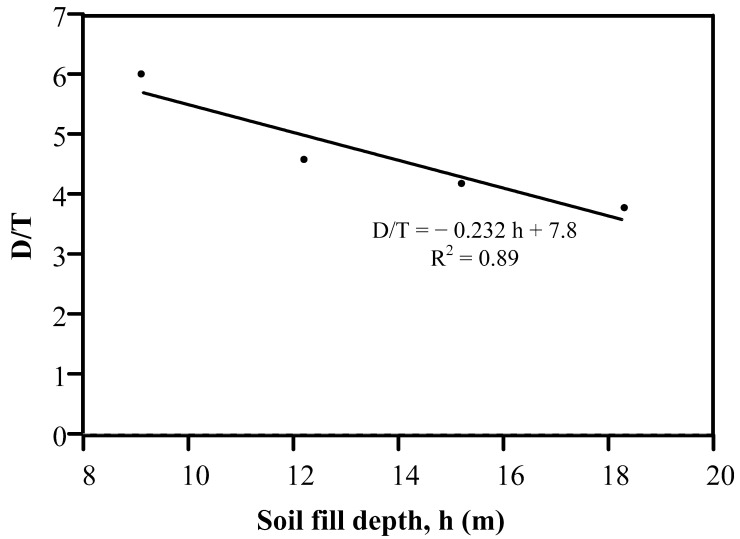
Soil fill depth versus optimum D/T ratio.

Figure 8 illustrates the average optimum D/T ratio for various soil fill depth values; the data were based on the last column in Table 6. The figure reveals that the optimum D/T ratio linearly decreased with the soil fill depth, and the optimum D/T could be related to the soil depth using Equation (1). This equation is very important for pipeline designers to select the optimum pipeline geometry for various soil fill depths.

D/T ratio = −0.232 × soil fill depth (m) + 7.8(1)

### 4.4. Designers Aid in Selecting Optimum RC Pipeline Thickness

**Figure 9:**
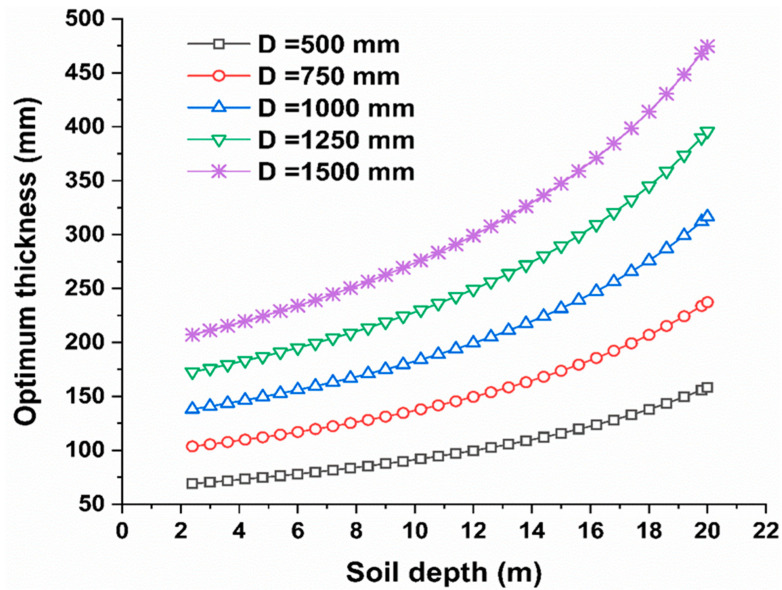
Optimum pipeline thickness under various soil fill depths and pipeline diameters.

This section includes a practical example to assist designers and practitioners in benefitting from this study and ensuring that the findings are generalizable. The example predicted the best thickness for pipes with diameters of 500, 750, 1000, 1250, and 1500 mm, with soil fill depths ranging from 2.4 m to 20 m taken into account, as shown in Figure 9. The optimal pipeline thickness was determined using Equation (1). According to Figure 9, the ideal thickness was determined by the pipe diameter and soil fill depth. For the examined pipe diameters up to 10 m, the optimal reinforced concrete pipe thickness was directly proportional to the soil depth.

However, beyond 10 m of soil depth, the ideal pipeline thickness was nonlinearly related to the soil depth, with the increasing rate in this stage (i.e., for soil depth greater than 10 m) being more significant than the slope of the first stage (i.e., for soil depth less than 10 m). Furthermore, the ideal thickness for a pipeline diameter of 500 mm at a soil depth of 2.4 m was 70 mm, while the optimum thickness for the soil fill when raised to 20 m was 160 mm. However, for pipe diameters of 1500 mm, the optimal thickness was 210 mm and 475 mm at soil depths of 2.4 and 20 m, respectively, indicating that the optimum thickness was much higher for big pipe diameters under a high burial soil depth. For example, the D/T ratio for a 1500-mm pipe diameter at a soil depth of 20 m was 1500 mm/475 mm = 3.15, but the D/T ratio at a soil depth of 2.4 m was 1500 mm/210 mm = 7.14. As previously indicated in Figure 8, the D/T ratio had an inverse relationship with the soil fill depth. The same holds for pipe diameters of 500, 750, 1000, 1250, and 1500 mm, where the optimal D/T decreased from 7.14 to 3.15 for soil depths of 2.4 and 20 m, respectively. As a result, regardless of the pipe diameter, the optimal pipe thickness rose (nearly doubles) as the soil depth increases from 2.4 m to 20 m. This example shows how designers and practitioners utilize Equation (1) to determine the best reinforced concrete pipeline for a given pipe geometry and soil depth. Thus, in construction projects, providing decision-support tools using machine and deep learning approaches is vital [37,38,39,40,41,42,43,44,45,46,47,48].

## 5. Summary and Conclusions

The current practice does not include guidelines for using the most efficient reinforced concrete pipeline geometry under deep soil embankments. To fill this gap and propose a guideline for designing reinforced concrete pipelines, this study investigated the performance of reinforced concrete pipelines of various diameters buried under soil fill depths of 9.1 to 18.3 m. High costs accompany testing or inspecting pipelines. Thus, a tool such as finite element analysis is critical for reducing such costs; CANDE 2019 software was used here as the FEA tool to conduct the parametric study. As a result, 58 FEA runs were executed, whereas the flexural steel reinforcement (FSR) and the maximum pipeline deflection in each FEA run were reported. In addition, the optimum pipeline diameter-to-thickness (D/T) ratio that had the least required FSR and crack width did not exceed 0.25 mm, which was specified for various pipeline diameters and soil fill depths. The Duncan/Selig soil parameters SW90 and SW95 were used to define the soil properties for the in situ bedding and fill layers. The analysis results revealed that the optimum reinforced concrete pipeline diameter-to-thickness (D/T) ratio was 6.0, 4.6, 4.2, and 3.8 for soil fill depths of 9.1, 12.2, 15.2, and 18.3 m, respectively. In addition, the deflection range of the investigated reinforced concrete pipelines was between 0.5 to 13 mm, which indicates that the finite element analysis carefully selected the pipeline thickness, required FSR, and concrete crack width. In contrast, the pipeline did not undergo excessive deformation.

A detailed example was presented to help designers and practitioners select the optimum thickness for various soil depths (2.4 to 20 m) and different pipeline diameters (500, 750, 1000, 1250, and 1500 mm), which ensures the generalization of the proposed equation for a variety of pipeline conditions. The study proposed an equation to select the optimum diameter-to-thickness ratio for various soil fill depths, which will help practitioners select cost-effective pipeline geometry and steel reinforcement at a specific soil fill depth. The optimum pipeline thickness was significantly sensitive to the soil fill depth. In contrast, the optimum reinforced concrete pipeline thickness was substantially increased (almost doubled) when the soil fill depth increased from 2.4 to 20 m. Field data collection of pipelines is required for further research to reach a solid stage in order for more analytical investigations. It is thus recommended to use deep and machine learning algorithms to improve the analysis accuracy and pave the road for researchers to deeply validate the proposed model performance.

## Figures and Tables

**Table 1:** Dimensions of the pipes used in the analysis.

Pipe Designation	Pipe 24	Pipe 36	Pipe 48	Pipe 60
Pipe diameter, mm (in.)	610 (24)	915 (36)	1220 (48)	1524 (60)

**Table 2:** Thickness and soil fill depth of the analyzed reinforced concrete pipes.

Soil Fill Height h, m (ft)	Reinforced Concrete Pipe Thickness mm (in)			
	Pipe 24	Pipe 36	Pipe 48	Pipe 60
9.1 (29.9)	102 (4)	102 (4)	102 (4)	109 (4.3)
	152 (6)	152 (6)	152 (6)	152 (6)
	203 (8)	203 (8)	203 (8)	203 (8)
	--	--	254 (10)	254 (10)
12.2 (40)	102 (4)	114 (4.5)	102 (4)	152 (6)
	152 (6)	152 (6)	152 (6)	254 (10)
	203 (8)	203 (8)	178 (7)	305 (12)
	--	--	254 (10)	--
15.2 (49.9)	102 (4)	203 (8)	190 (7.5)	152 (6)
	152 (6)	254 (10)	305 (12)	215 (8.5)
	203 (8)	312 (12.3)	363	254 (10)
	--	--	--	317 (12.5)
	--	--	--	363 (14.3)
18.3 (60)	89 (3.5)	152 (6)	152 (6)	152 (6)
	127 (5)	254 (10)	203 (8)	210 (8.3)
	152 (6)	312 (12.3)	254 (10)	262 (10.3)
	203 (8)	--	284 (11.2)	318 (12.5)
	--	--	363 (14.3)	363 (14.3)

**Table 3:** Duncan/Selig soil parameters used in the FEA analysis.

Parameter	Parameter Symbol	Soil Properties	
		SW95 (Fill Soil)	SW90 (Bedding)
Young’s tangent	*K* (kPa)	950	640
Modulus parameters	*n*	0.6	0.43
	*C* (kPa)	0	0
	*ϕ*_0_ (Degree)	48	42
	Δ*ϕ* (Degree)	8	4
	*R_f_*	0.7	0.75
Bulk parameters	*B_i_/P_a_*	74.8	40.8
	ε_u_	0.02	0.05
Soil unit weight	γ (kN/m^3^)	18.9	18.9

Note: *K* = modulus number; *n* = modulus exponent; *R_f_* = failure ratio; *C* = cohesion; *ϕ*_0_ = initial internal friction angle; Δ*ϕ* = reduction in friction angle; *B_i_* = initial tangent bulk modulus; *P_a_* = atmosphere pressure; and ε_u_ = ultimate volumetric strain.

**Table 4:** Concrete and steel reinforcement properties.

Concrete Properties	
Unit weight (kN/m^3^)	23.6
Compressive strength (MPa)	34.5
Young’s modulus (MPa)	29,557
Poison’s ratio	0.17
**Steel Reinforcement Properties**	
Yield stress (MPa)	448
Young’s modulus (MPa)	200,000
Poison’s ratio	0.3
Concrete cover to the centerline of steel rebar cage, mm	32

**Table 5:** The optimum thickness of the analyzed reinforced concrete pipes.

Soil Depth (m)	The Optimum Thickness of the Reinforced Concrete Pipe (mm)			
	Pipe 24	Pipe 36	Pipe 48	Pipe 60
9.1	102	152	203	254
12.2	152	203	254	304
15.2	152	203	305	363
18.3	203	254	284	363

**Table 6:** Optimum diameter-to-thickness ratio (*D*/*T*) of the analyzed reinforced concrete pipes.

Soil Depth (m)	The Optimum *D/T* Ratio of the Reinforced Concrete Pipe				Average *D/T*
	Pipe 24	Pipe 36	Pipe 48	Pipe 60	
9.1	6.0	6.0	6.0	6.0	6.0
12.2	4.0	4.5	4.8	5.0	4.6
15.2	4.0	4.5	4.0	4.2	4.2
18.3	3.0	3.6	4.3	4.2	3.8
